# The Disordered C-Terminus of Yeast Hsf1 Contains a Cryptic Low-Complexity Amyloidogenic Region

**DOI:** 10.3390/ijms19051384

**Published:** 2018-05-06

**Authors:** Jordi Pujols, Jaime Santos, Irantzu Pallarès, Salvador Ventura

**Affiliations:** 1 Institut de Biotecnologia i de Biomedicina, Universitat Autònoma de Barcelona, E-08193 Bellaterra (Barcelona), Spain; jordi.pujols@uab.cat (J.P.); jaime.santos@uab.cat (J.S.); 2 Departament de Bioquímica i Biologia Molecular, Universitat Autònoma de Barcelona, E-08193 Bellaterra (Barcelona), Spain

**Keywords:** amyloid, Hsf1, protein aggregation, intrinsically disordered regions, Q/N-rich regions, low complexity, molecular recognition features

## Abstract

Response mechanisms to external stress rely on networks of proteins able to activate specific signaling pathways to ensure the maintenance of cell proteostasis. Many of the proteins mediating this kind of response contain intrinsically disordered regions, which lack a defined structure, but still are able to interact with a wide range of clients that modulate the protein function. Some of these interactions are mediated by specific short sequences embedded in the longer disordered regions. Because the physicochemical properties that promote functional and abnormal interactions are similar, it has been shown that, in globular proteins, aggregation-prone and binding regions tend to overlap. It could be that the same principle applies for disordered protein regions. In this context, we show here that a predicted low-complexity interacting region in the disordered C-terminus of the stress response master regulator heat shock factor 1 (Hsf1) protein corresponds to a cryptic amyloid region able to self-assemble into fibrillary structures resembling those found in neurodegenerative disorders.

## 1. Introduction

Eukaryotic cells have the capacity to overcome detrimental environmental conditions, such as hyperthermia or oxidative stress. This ability to maintain a balanced proteostasis depends on a restricted group of polypeptides, named heat shock proteins (HSPs) [1,2]. This set of protective proteins are designed to guarantee protein solubility and impede a massive loss of protein function due to degradation, post-transcriptional modifications, or aggregation. In this context, heat shock factor 1 (Hsf1) has been classically considered as the master orchestrator of this finely tuned response [3]. Hsf1 is an inducible transcription factor that, after activation, leads to the transcription of several HSPs and the subsequent metabolic readjustment [4]. Generically, in order to execute its function, Hsf1 undergoes trimerization, nuclear specific localization, and an extensive range of post-transcriptional modifications [2]. Indeed, under basal conditions, the protein is subjected to a negative regulation loop in which intramolecular contacts and specific interactions with other HSPs such as HSP90 preserve the inactive monomeric form of the protein [2,5,6].

Hsf1 function is conserved among different species, the DNA binding domain (DBD) being the most conserved region in the protein sequence. Despite the fact that the DBD corresponds to a folded domain, Hsf1 is thought to be mostly unstructured [7]. In fact, it is well-established that numerous proteins related with DNA-binding and transcriptional regulation lack ordered tertiary structure or contain long disordered segments under physiological conditions [8,9]. In these proteins, disordered regions contribute to their conformational plasticity and client promiscuity and this is crucial to establish a wide range of specific interactions [10]. In many cases, these interactions seem to depend on the existence of short sequence motifs which may or may/not undergo a disorder to order transition upon binding [11,12,13,14]. In addition, it is becoming increasingly evident that the compositional bias of certain disordered low complexity regions is an important determinant of protein–protein interactions in transcription factors [15].

It has been shown that the physicochemical properties behind functional protein–protein interactions overlap significantly with those accounting for the anomalous interactions leading to protein aggregation [16]. As a result, interaction sites in protein complexes [17,18], as well as the interfaces of homomeric and heteromeric globular proteins, display significant amyloidogenic propensity [19,20]. This implies that exposed aggregation-prone regions cannot be totally purged out by evolution from the sequences of globular proteins, because they are needed for binding and/or functional assembly [18,19]. One question is whether in disordered proteins, where the bulk of the sequence is exposed to solvent, this duality between the establishment of functional and deleterious interactions also occurs. It is shown here that, in yeast Hsf1, a disordered and low complexity region at the C-terminus of this transcription factor is predicted to act in molecular recognition, at the cost of aggregating spontaneously into ordered amyloid assemblies.

## 2. Results

### 2.1. Identification of a Cryptic Amyloid Sequence Inside a Disordered and Low Complexity Region in Hsf1

**Figure 1:**
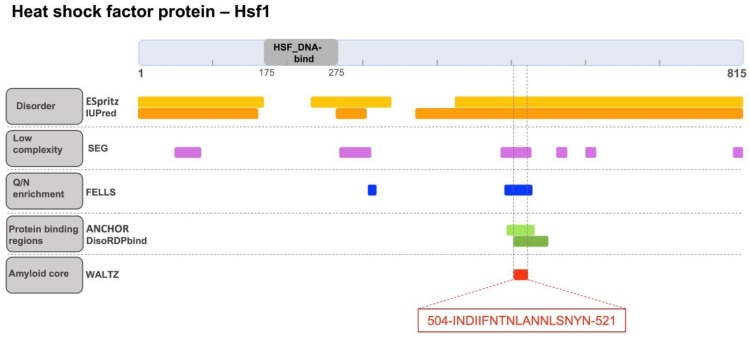
The cryptic amyloid core of heat shock factor 1 protein (Hsf1) in *Saccharomyces cerevisiae* (*S. cerevisiae*). Hsf1 diagram showing the location of the identified Pfam domain (grey), predicted disordered regions (orange), low complexity segments (violet), glutamine/asparagine (Q/N)-rich regions (blue), protein binding sites (green), and the cryptic amyloid core (red). The sequence of the amyloid core is shown in the red box. Hsf1: heat shock protein 1.

The order/disorder content of yeast Hsf1 was studied. First, the locations of constituent functional domains and unstructured regions in the full-length protein were examined (Figure 1). PFAM indicates that yeast Hsf1 contains a single annotated functional domain comprising residues 175–275, which correspond to the HSF_DNA-binding domain [21]. This domain is highly conserved both structurally and sequentially from yeast to humans and binds to specific DNA regulatory regions involved in the modulation of the complex HSF transcriptional activity [2]. Thus, according to PFAM, less than 15% of Hsf1 residues are implicated in the formation of functional folded moieties in the monomeric state. Indeed, the N-terminal Hsf1 1–167 region has been experimentally shown to lack any regular structure [22]. This is consistent with the predictions of protein disorder provided by two different algorithms, ESpritz-NMR [23] and IUPred [24], which point out that the protein regions at the two sides of the HSF_DNA-binding domain are essentially disordered (Figure 1). The SEG algorithm [25] indicates that Hsf1-disordered regions contain several short low complexity segments (Figure 1). We used FELLS [26] to determine if any of these low complexity regions displayed compositional bias. Interestingly enough, the region comprising residues 493–527 exhibits a striking compositional bias, with an overall asparagine content of 40% (Figure 1). Notably, this is an N enrichment comparable to that observed in the prion domains of several yeast prions [27,28]. In yeast, these glutamine/asparagine (Q/N)-rich domains promote self-assembling interactions, which result in conformational conversion [29] and phase separation [30].

We have recently shown that the disordered, low complexity, Q/N-rich regions of yeast prions and other prion-like proteins might contain cryptic amyloidogenic regions that can contribute significantly to trigger protein assembly [31,32,33]. These regions differ from the classical amyloid cores found in pathogenic proteins in that they are longer and more polar, in such a way that the amyloid potential is less concentrated, allowing the protein to remain in a soluble state under most physiological conditions, while still keeping a certain amyloid propensity that might facilitate its assembly in certain circumstances [34]. We analyzed whether the disordered and N-rich Hsf1 region 493–527 hid an amylogenic stretch using WALTZ, an algorithm that exploits a position-specific scoring matrix to identify potential amyloid sequences [35]. WALTZ predicted the 504-INDIIFNTNLANNLSNYN-521 sequence as the longest region displaying significant amyloid potential. It is worth mentioning that, in this segment, 55% of the residues have a polar nature and accordingly, the hydropathicity is much lower than that of the typical amyloid cores found in disease-linked proteins (Table 1). This explains why well-validated aggregation predictors like Aggrescan [36] and Zyggregator [37], which consider sequence hydrophobicity as a major contributor to protein aggregation, did not detect any aggregation-prone regions in Hsf1 sequence 493–527.

Here, it was investigated whether our candidate amyloid core could potentially be a region involved in the establishment of intermolecular contacts. To this end, ANCHOR [38] and DisoRDPbind [39] were used, which are two conceptually different algorithms that aimed to identify protein binding sequences in disorder proteins or regions. Interestingly enough, both programs successfully identified the sequence stretch around Hsf1 residues 504–521 as a potential protein binding region (Figure 1).

Overall, the bioinformatics analysis suggest that Hsf1 might contain a functional amyloid core embedded in a disordered and low complexity region. To confirm that this soluble transcription factor bears a sequence with the potential to form amyloid-like assemblies, the 18-residue-long peptide was synthesized and characterized experimentally.

### 2.2. The Amyloid Core of Hsf1 Assembles into β-Sheet Enriched Aggregates

**Figure 2:**
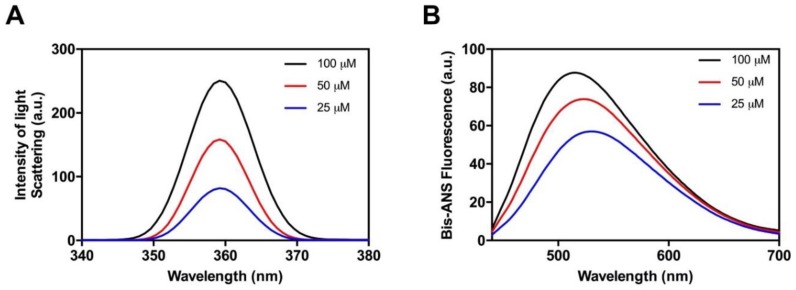
Aggregation of the soft amyloid core in Hsf1 as a function of the concentration. Aggregation (**A**) and the formation of hydrophobic surfaces (**B**) were monitored by light scattering and 4,4-bis-1-anilinonaphthalene-8-sufonate (Bis-ANS) fluorescence emission, respectively, at various peptide concentrations.

As a first step, and in order to determine whether the candidate peptide has the ability to aggregate in vitro, Hsf1 amyloid core peptide samples were prepared at 25, 50, and 100 µM and incubated at 25 °C for 120 h. Aggregation was systematically monitored by measuring changes in synchronous light scattering [41]. As shown in Figure 2A, a concentration-dependent scattering signal could be observed, reaching a maximum at 100 µM of peptide. These data indicate that despite the candidate amyloid core is clearly less hydrophobic than those in classic amyloids, it can still establish the initial intermolecular interactions that lead to the buildup of high-order assemblies. 

Next, we explored the presence of exposed hydrophobic clusters in the formed peptide aggregates by measuring their binding to 4,4-bis-1-anilinonaphthalene-8-sufonate (Bis-ANS), a dye that exhibits fluorescence enhancement upon interaction with exposed hydrophobic surfaces as well as a blue shift of the peak maximum [42,43] (Figure 2B). We found complete agreement between the Bis-ANS and the light-scattering assays. The Bis-ANS fluorescence presents a concentration-dependent intensity increase and the maximum emission shifts from 521 nm (in the presence of 25 µM of peptide) to 505 nm at 100 µM. These spectral changes indicate that the peptide aggregation results in the formation of novel/larger hydrophobic patches, thus suggesting that the interactions between the peptide hydrophobic residues lead to its assembly.

**Figure 3:**
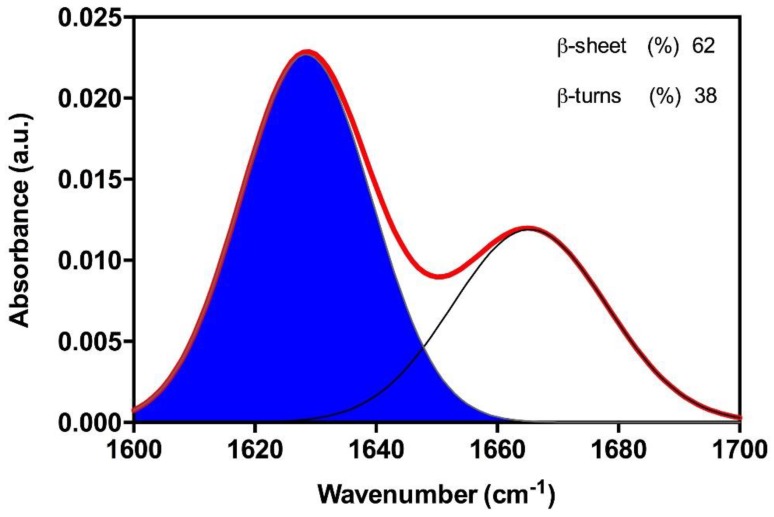
Amyloid core secondary structure of Hsf1. The secondary structure was determined from the Attenuated Total Reflectance (ATR) Fourier Transform Infrared (FT-IR) absorbance spectrum in the amide I region. The red line corresponds to the original spectrum; the blue area indicates the contribution of the inter-molecular β-sheet signal to the total area upon Gaussian deconvolution.

To analyze the secondary structure content of the Hsf1 peptide aggregates, the amide I region of the Fourier Trasform Infrared (FT-IR) spectrum (1700–1600 cm^−1^) upon its incubation at 100 µM for 120 h was recorded. Deconvolution of the obtained spectra allow us to identify a strong band at 1628 cm^−1^, which is indicative of a predominance of extended intermolecular β-sheet structures. This signal is the largest contributor to the absorbance spectrum, accounting for 62% of the total area. The other detected peak at 1667 cm^−1^ is attributable to the presence of β-turns (Figure 3). Because no anti-parallel β-sheet band was detected at ~1690 cm^−1^, a preferential parallel β-sheet organization for the self-assembled peptide is suggested.

Collectively, the data indicate that the predicted amyloid core of Hsf1 spontaneously self-assembles into highly ordered supramolecular structures highly enriched in β-sheet content.

### 2.3. Hsf1 Amyloid Core Forms Amyloid-Like Fibrillary Structures

**Figure 4:**
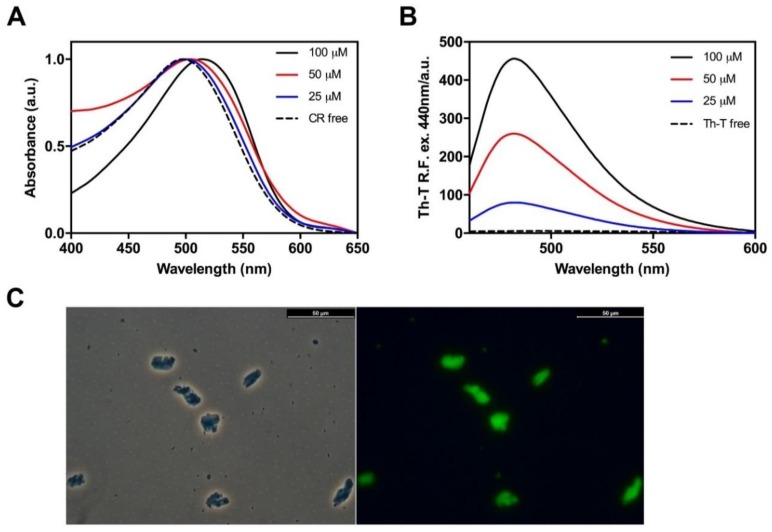
Binding of amyloid dyes to the cryptic amyloid core of Hsf1. (**A**) Congo red (CR) spectral changes in the presence of Hsf1 peptide incubated at various concentrations during 120 h at 25 °C; note the characteristic shift from 497 nm to 515 nm when the dye is bound to amyloid-like aggregates; (**B**) changes in the fluorescence emission spectrum of Thioflavin-T (Th-T) when excited at 440 nm upon binding to the aggregated peptide at various concentrations after 120 h incubation at 25 °C; (**C**) Thioflavin-S fluorescence of stained aggregated amyloid material of Hsf1 at 100 μM in Phosphate buffered saline (PBS) buffer after 120 h incubation at 25 °C. Images were obtained at 40× magnification by phase contrast and fluorescence microscopy displaying the green fluorescence characteristic of amyloid structures.

Next, to assess whether the detected β-sheets structures in Hsf1 peptide aggregates correspond to amyloid-like assemblies, the amyloid-specific dyes Congo red (CR), Thioflavin-T (ThT) and Thioflavin-S (ThS) were used [44]. CR is widely used for the detection of amyloid material [45]; when the dye is bound to cross β-sheet structures there is hypercromicity and a red shift of the absorbance maximum. We analyzed CR binding to the aggregated peptide at 25, 50, and 100 µM. As expected, the peptide at 100 µM promoted the maximum shift in the CR spectra from 497 nm, in the absence of peptide, to 515 nm (Figure 4A).

Similarly, binding of ThT to the Hsf1 amyloid core peptide induced a large enhancement in the intensity of ThT fluorescence emission relative to the free dye, increasing as a function of the peptide concentration (Figure 4B). Additionally, binding of ThS to 100 µM peptide aggregate was tested by fluorescence microscopy. Areas rich in fibrous material appear stained with ThS, giving a bright green-yellow fluorescence against a dark background (Figure 4C). Overall, the results reported from the dye staining experiments strongly suggest an amyloid-like organization of the peptide in the aggregates.

**Figure 5:**
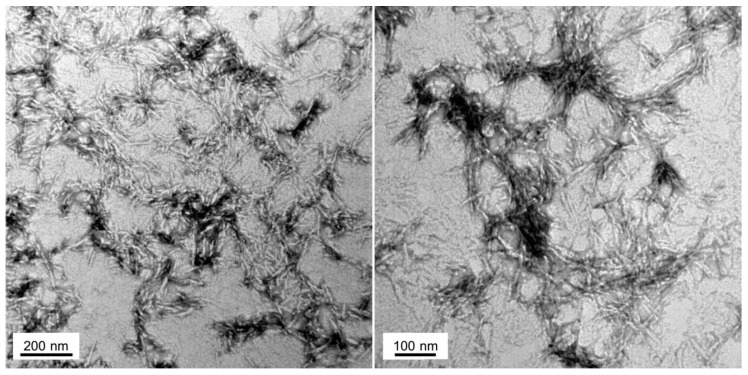
Fibrillar structure of Hsf1 cryptic amyloid core. Representative electron micrographs of Hsf1 aggregated peptide at 100 μM in PBS after 120 h of incubation at 25 °C.

To check whether the peptide assemblies could have any internal fibrillar structure, we performed a morphological analysis using transmission electron microscopy (TEM) (Figure 5). In agreement with the above reported results, the 100 µM Hsf1 amyloid core peptide solution self-assembles, forming fibrillar arrangements after 120 h incubation at 25 °C. The resultant fibrils are straight, unbranched, and exhibit a diameter that varies from 6 to 12 nm, being thus similar to the fibrillar structures formed by amyloidogenic proteins involved in neurodegenerative disorders [46].

### 2.4. Hsf1 Amyloid Core Self-Assembles Very Fast

**Figure 6:**
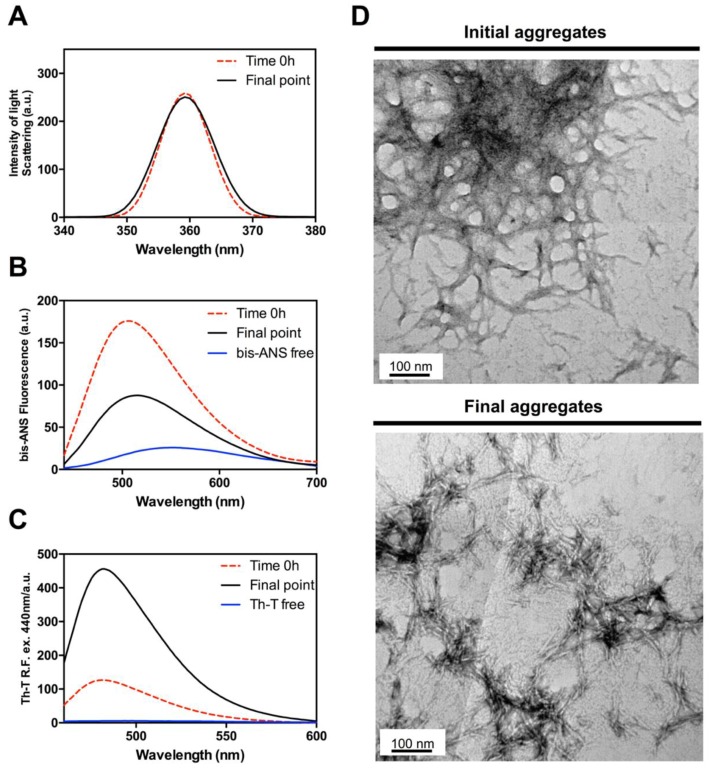
Evolution of the amyloid core of Hsf1 from initial non-ordered aggregates into mature amyloid fibrils. (**A**) Light scattering; (**B**) Bis-ANS fluorescence emission; (**C**) ThT fluorescence emission; (**D**) representative TEM images of initial and final Hsf1 peptide aggregates. The final point corresponds to 120 h.

While the classical predictors suggested a low amyloid propensity for the Hsf1 peptide, the above data indicate the contrary. To assess if the aggregation tendency of the peptide was high enough to promote its immediate assembly, the properties of the solution upon peptide dilution at 100 µM in Phosphate buffered saline (PBS) (time 0 h) were monitored, and compared with those of the final aggregates (120 h). Synchronous light scattering indicated an initial level of aggregated material similar to that observed at the end point of the reaction (Figure 6A). However, the conformational properties of early and late aggregates are significantly different. Early aggregates display a higher Bis-ANS signal (Figure 6B); in contrast, they exhibit much lower ThT binding than late aggregates (Figure 6C). This observation is consistent with their less ordered architecture as assessed by TEM images, which show the presence of proto-fibrillar assemblies that tend to coalescence in larger aggregates (Figure 6D). Altogether, the data indicate that the Hsf1 amyloid core undergoes a very fast aggregation reaction into proto-fibrils with exposed hydrophobic patches and a low average content of amyloid-like regions. The evolution of these aggregates into the typical amyloid assemblies observed at the end of the reaction necessarily involves the burial of a fraction of previously exposed hydrophobic residues inside the ordered fibrils. These data corroborate the strong aggregation propensity of the identified disordered region of Hsf1.

## 3. Discussion

The present work exemplifies a strategy to uncover hidden amyloid segments in the disordered regions of proteins. Together, the computational and biophysical analyses provide evidence for the existence of a cryptic amyloid core embedded in a disordered and low complexity region of yeast Hsf1. A peptide corresponding to this region spontaneously self-assembles into CR- and ThT-positive β-sheet enriched amyloid fibrils in vitro.

While hydrophobic interactions seem to be important for the assembly, the majority of the residues in the amyloid core have a polar nature, which endorses this segment with an overall lower hydrophobicity than the cores in pathogenic amyloids. Indeed, 70% of the polar residues in this region correspond to asparagine, a composition that is reminiscent of that found in the amyloidogenic regions of prion-like domains, which contribute significantly to their conformational conversion [28,31]. While in the light of the extensive literature on Hsf1 it is unlikely that in vivo and in the context of the full-length protein this region would drive Hsf1 amyloid formation, it is now evident that protein evolution tends to purge out aggregation-prone regions if they do not serve functional purposes, especially if they are constantly exposed to solvent, as occurs in disordered protein regions [47]. In fact, in contrast to classical pathogenic amyloids, when amyloid sequences occur in disordered regions, their aggregation potential tends to be less concentrated, thus precluding uncontrolled aggregation and suggesting they might play instead a functional role [33]. Indeed, ANCHOR [38] and DisoRDPbind [39] predictions converge to suggest that the cryptic amyloid core we identified in Hsf1 might correspond to a molecular recognition element [48]. If this comes to be true, it would imply that both in the context of folded and disordered proteins the determinants for functional and deleterious interactions tend to overlap. Indeed, a number of diseases that have been associated with disorder-containing proteins seem to be caused by undesirable protein interactions [49].

This work contributes new data in favor of the hypothesis that protein aggregation-prone regions might play a role in protein–protein interactions [50]; thus supporting the vision that, despite the medical relevance of pathogenic amyloid formation, this deleterious reaction constitutes only one of the many different aggregation-driven phenomena that might occur in a cell [51].

It is evident that more work is needed to confirm the suggested relationship between binding and amyloid propensities in the context of Hsf1. However, the fact that the properties of the identified amylogenic segment coincide precisely with those of the amyloid core we found previously in the prion-like domain of the bacterial transcription terminator Rho [52,53], which was afterwards confirmed to self-assemble in vivo through this specific region in order to regulate its activity [54], indicates that a deeper characterization of Hsf1 conformational and self-assembling properties will be worth the effort.

## 4. Materials and Methods

### 4.1. Materials

The 18-residue peptide with the sequence INDIIFNTNLANNLSNYN, that corresponds to the predicted soft amyloid core of S. cerevisiae Hsf1, was purchased from CASLO ApS (Scion Denmark Technical University, Copenhagen, Denmark) and used without further purification. Other chemicals reagents and buffers were purchased from Sigma-Aldrich (Sigma Aldrich, Merck KGaA, Darmstadt, Germany).

### 4.2. Computational Identification of Hsf1 Soft Amyloid Cores in Low Complexity Regions

The sequence of *Saccharomyces cerevisiae* (*S. cerevisiae*) Hsf1 (P10961) was downloaded from Uniprot [55]. It was first scanned for the presence of regions with a compositional bias with at least 30% of Q/Ns inside intrinsically disordered regions using FELLS [26] and IUPred algorithms [24,35]. The identified region (493–527), was further evaluated in a search for soft amyloid cores with WALTZ [35], which resulted in the identification of one positive candidate region. A search for a protein binding region within this soft amyloid core was performed using ANCHOR [38] and DisoRDPbind [39], resulting in the identification of the Hsf1 soft amyloid core as a putative protein binding region.

### 4.3. Hsf1 Soft Amyloid Core Peptide Preparation

The peptide was initially resuspended in 1,1,1,3,3,3-hexafluoropropanol to remove any residual aggregate and afterwards the solvent was evaporated under vacuum. The peptide stock solution was prepared solubilizing the resulting peptide film at a final concentration of 5 mM in 100% dimethyl sulfoxide and stored at −80 °C. Before each analysis, the sample was diluted to 150 μM in PBS pH 7.4. For aggregation assays the peptide was diluted to 25, 50, and 100 µM and incubated for 120 h at 25 °C.

### 4.4. Synchronous Light Scattering

Synchronous light scattering was monitored using a Cary Eclipse spectrofluorometer (Varian, Palo Alto, CA, USA). Spectra were recorded as the accumulation of three consecutive scans and the conditions of the acquisition were: excitation wavelength of 360 nm, emission range from 340 to 380 nm, and slit widths of 5 nm at 25 °C in PBS pH 7.4 buffer. The samples were sonicated for 10 min in an ultrasonic bath (VWR ultrasonic cleaner, VWR International, Radnor, PA, USA) before measurement.

### 4.5. Bis-ANS Binding

The relative fluorescence spectra of 4,4-bis-1-anilinonaphthalene-8-sufonate (Bis-ANS, Sigma Aldrich, Merck KGaA, Darmstadt, Germany) were obtained using a Cary Eclipse spectrofluorometer (Varian, Palo Alto, CA, USA). Spectra were recorded as the accumulation of three consecutive scans and the conditions of the acquisition were: excitation wavelength of 370 nm, emission range from 400 to 700 nm, and slit widths of 5 nm; the measurements were performed at 25 °C in PBS buffer at pH 7.4. The samples were sonicated for 10 min in an ultrasonic bath (VWR ultrasonic cleaner) before dye addition. Prior to fluorescence measurements samples were incubated at room temperature for 5 min in the presence of a final Bis-ANS concentration 10 μM. A Bis-ANS solution without peptide was used as a control.

### 4.6. Attenuated Total Reflectance (ATR) Fourier Transform Infrared (FTIR) Spectroscopy

Attenuated total reflectance Fourier transform infrared (ATR FT-IR) spectroscopy analysis of peptide fibrils was performed using a Bruker Tensor FT-IR Spectrometer (Bruker Optics, Berlin, Germany) with a Golden Gate MKII ATR accessory. Aggregated peptide solution, previously sonicated for 10 min in an ultrasonic bath (VWR ultrasonic cleaner), was dried out under a N_2_ (g) atmosphere and each spectrum consisted of 16 independent scans, measured at spectral resolution of 2 cm^−1^ within the 1800–1500 cm^−1^ range. Spectral data were acquired and normalized using the OPUS MIR Tensor 27 software. The individual components of the spectrum were determined through second derivative analysis of the spectra and deconvoluted afterwards into overlapping Gaussian curves. The amplitude, mass center, bandwidth at half of the maximum amplitude, and area for each Gaussian function were calculated employing the nonlinear peak-fitting program PeakFit v4.12 (Systat Software Inc., San Jose, CA, USA). The PBS pH 7.4 buffer without peptide was used as a control and subtracted from the absorbance signal before deconvolution.

### 4.7. Binding to Amyloid Dyes

Congo red binding (CR) to aggregated peptide solutions was determined using a Cary 100 UV/Vis spectrophotometer (Varian, Palo Alto, CA, USA) in the 400–700 nm range using a 1 cm optical length quartz cuvette placed in a thermostated cell holder at 25 °C.

The samples were sonicated for 10 min in an ultrasonic bath (VWR ultrasonic cleaner) before dye addition. Prior to absorbance measurements samples were incubated at room temperature for 10 min in the presence of 5 µM final CR concentration. PBS pH 7.4 buffer with 5 μM CR and without peptide was used as a control.

The relative fluorescence spectra of Thioflavin-T (ThT, Sigma Aldrich, Merck KGaA, Darmstadt, Germany) were monitored using a Cary Eclipse spectrofluorometer (Varian, Palo Alto, CA, USA). Spectra were recorded as the accumulation of three consecutive scans and the conditions of the acquisition were: excitation wavelength of 440 nm, emission range from 460 to 600 nm, and slit widths of 5 nm; the measurements were performed at 25 °C in PBS buffer at pH 7.4. The samples were sonicated for 10 min in an ultrasonic bath (VWR ultrasonic cleaner) before dye addition. Prior to fluorescence measurements, samples were incubated at room temperature for 2 min in the presence of a 25 µM final ThT concentration to ensure that thermal equilibrium had been achieved. PBS buffer at pH 7.4 with 25 μM ThT and without peptide was used as a control.

For the Thioflavin-S (ThS, Sigma Aldrich, Merck KGaA, Darmstadt, Germany) staining by fluorescence microscopy, Hsf1 aggregates were incubated for 1 h at room temperature with continuous agitation at 80 rpm in the presence of ThS at a final concentration of 150 µM in PBS buffer at pH 7.4. Then, the excess of ThS was washed up by centrifugation and resuspension of the precipitated fraction in PBS buffer at pH 7.4 three times. Finally, the precipitated fraction was resuspended in a final volume of 10 μl, placed on a microscope slide, and the coverslip sealed. Images of the peptide aggregates bound to ThS were obtained at 40-fold magnification under UV light or using phase contrast in a Leica fluorescence microscope (Leica DMRB, Heidelberg, Germany).

### 4.8. Transmission Electron Microscopy (TEM)

An aliquot (5 μL) of 100 µM peptide incubated for 120 h at 25 °C and sonicated for 10 min in an ultrasonic bath (VWR ultrasonic cleaner) was placed on carbon-coated copper grids and allowed to absorb for 5 min. Then, the excess of solution was wicked away using small pieces of ashless filter paper; the samples were stained with 5 μL of uranyl acetate (2% w/v) for 2 min for the negative staining. Finally, the excess of uranyl acetate was removed as above, and the grids left to dry before the TEM observation. Amyloid fibrils images were obtained using a JEOL JEM-1400 electron microscope, (JEOL Peabody, MA, USA) operated at an 80-kV accelerating voltage.

## Figures and Tables

**Table 1:** Amyloid core hydropathicity of Hsf1 in *S. cerevisiae*. The GRAVY score (average hydrophobicity and hydrophilicity) was evaluated using the EXPASY ProtParam tool [40]. The GRAVY scores for the amyloid cores of α-synuclein (ASYN) and Aβ42, involved in Parkinson’s and Alzheimer’s disease, respectively, are shown for comparison.

Protein	Amyloid Core	Gravy Score
Hsf1	INDIIFNTNLANNLSNYN	−0.283
ASYN	GVLYVG	1.683
	GGAVVTGVTAVAQ	1.238
Aβ42	GAIIGLMVGGVVI	2.462
	QKLVFFAE	0.562
